# Patterns of chemotherapy use with primary radiotherapy for localized bladder cancer in patients 65 or older

**DOI:** 10.3389/fonc.2024.1341655

**Published:** 2024-05-15

**Authors:** Fady Ghali, Sarah K. Holt, Elizabeth L. Koehne, Jonathan J. Chen, Emily S. Weg, Jay J. Liao, Jing Zeng, Petros Grivas, Jessica E. Hawley, Andrew C. Hsieh, Robert Bruce Montgomery, Jonathan L. Wright

**Affiliations:** ^1^ Department of Urology, Yale School of Medicine, New Haven, CT, United States; ^2^ Department of Urology, University of Washington School of Medicine, Seattle, WA, United States; ^3^ Division of Radiation Oncology, Department of Medicine, University of Washington School of Medicine, Seattle, WA, United States; ^4^ Division of Hematology Oncology, Department of Medicine, University of Washington School of medicine, Seattle, WA, United States; ^5^ Fred Hutchinson Cancer Center, Seattle, WA, United States

**Keywords:** bladder cancer, organ-preservation, radiation, chemotherapy, trimodal therapy

## Abstract

**Introduction:**

Bladder preservation with concurrent chemoradiotherapy after maximum transurethral resection of bladder tumor is an alternative to radical cystectomy in select patients with muscle invasive bladder cancer (MIBC). Concurrent administration of radio-sensitizing chemotherapy and radiation therapy (RT) has been shown to have superior disease control compared with RT alone and can often be administered with modest added toxicity. We sought to describe national patterns of chemotherapy use.

**Methods:**

The linked surveillance, epidemiology, and end results (SEER)-Medicare database was used to identify patients with cT2-4, N0/X, M0/X BC who received radiation between 2004 and 2018. Data on demographics, clinicopathologic factors, therapy and outcomes were extracted. Concurrent utilization of chemotherapy with RT was also identified (CRT). Multivariate logistic regression (MVA) models were used to explore factors associated with receipt of chemotherapy and overall survival (OS).

**Results:**

2190 patients met inclusion criteria. Of these, 850 (38.8%) received no chemotherapy. Among those receiving chemotherapy, the most frequent regimens were single agent carboplatin, cisplatin, or gemcitabine. Factors that were independently associated with decreased likelihood of chemotherapy use were increasing age (OR 0.93, CI 0.92 – 0.95), Hispanic race (compared with White, OR 0.62, CI 0.39 – 0.99), cT3 or T4 (compared with cT2, OR 0.70, CI 0.55 – 0.90), and lower National Cancer Institute comorbidity index (OR 0.60, CI 0.51 – 0.70) (p < 0.05). Variables independently associated with increased likelihood of receipt of chemotherapy were married status (OR 1.28, CI 1.06 – 1.54), higher socioeconomic status (OR 1.31, CI 1.06 – 1.64), and later year of diagnosis (OR 1.09, CI 1.06 – 1.12). Receipt of concurrent chemotherapy with RT was associated with superior OS compared with RT alone.

**Conclusion:**

Over a third of patients >/65 years old receiving curative-intent RT for MIBC do not receive concurrent chemotherapy. Considering the improvement in oncologic outcomes with CRT over RT alone and more options, such as low dose gemcitabine which can be administered with modest toxicity, efforts are needed to identify barriers to utilization and increase the use of radio-sensitizing chemotherapy.

## Introduction

Radical cystectomy (RC) and pelvic lymph node dissection, ideally after neoadjuvant chemotherapy (NAC) in fit patients, is considered the gold standard therapy for localized resectable muscle-invasive bladder cancer (MIBC) in the United States (1, 2). The combination of chemotherapy and radiation therapy (CRT) after maximum transurethral resection of bladder tumor (TURBT), however, offers a bladder-preservation alternative for select patients who are unfit or unwilling to undergo RC. Despite significant efforts (3), no head-to-head randomized clinical trials between RC and CRT exist, yet observational data suggest comparable outcomes with those two treatment strategies are achievable in well-selected patients with MIBC (4–7). One recent review of ten studies, for example, identified equivalent 5-year overall and cancer-specific survival between the two modalities (8). Use of CRT as a primary treatment for MIBC is increasing in the United States, reflecting the desire of both patients and providers for organ-sparing treatment options (9).

Strong evidence supports the addition of concurrent systemic chemotherapy to primary radiation therapy (RT) in MIBC (10–13). Nonetheless, prior reports have documented poor utilization of chemotherapy for these patients (14, 15). While national guidance exists regarding choice of chemotherapeutic agents (1), significant heterogeneity has also been reported in institutional series. Further, studies show that certain chemotherapy agents, such as carboplatin, may be inferior to others for CRT (11). However, there is lack of high-level evidence from randomized phase III trials comparing different radio-sensitizing chemotherapy agents. Cancer registry studies have highlighted the low utilization of CRT, although such studies have been limited in defining the specific chemotherapy agents utilized (14). In fact, little is known regarding national patterns of chemotherapy selection with CRT use in MIBC. Additionally, the interactions of these treatment decisions with social determinants of health remain poorly understood; that remains a need aiming to address the important issue of healthcare disparities in the United States (US) and globally.

We sought to describe national patterns of chemotherapy utilization with primary RT with the aim of identifying potential avenues for improving care delivery for patients with localized MIBC. We hypothesize an increasing utilization of chemotherapy over time, and wide heterogeneity regarding the choice of the chemotherapy agent.

## Methods

### Data source

For the present study, we utilized the Surveillance, Epidemiology, and End Results (SEER)-linked Medicare database, a clinical database funded by the National Cancer Institute, which captures granular demographic, clinicopathologic data for 30-35% of the US population (16). Medicare linkage provides important access for medication administration and healthcare utilization.

### Study population

The study cohort included patients 65 years of age or older with diagnosis of MIBC (clinical stage T2-T4, urothelial, or transitional cell, carcinoma) by utilizing the International Classification of Diseases (ICD), 9^th^ and 10^th^ revisions, from January 1, 2004 to December 31, 2018. Patients with nodal metastasis, distant metastasis, and prior chemotherapy or radiation therapy were excluded.

Primary/definitive radiation was defined to be curative intent if it involved more than 10 fractions. We utilized procedure codes and Current Procedural Terminology (CPT) codes to identify patients that received RT with or without chemotherapy (**Supplementary Table 1**). This study was exempted by the institutional review board due to its nature.

### Data collection and coding

Our primary outcome was receipt of chemotherapy concurrently administered with RT. Secondary outcomes assessed were choice of agent and overall survival.

Variables abstracted included captured potential confounding variables, such as demographic factors (age, sex, race/ethnicity, median household income, socioeconomic status [SES]), clinical factors (diagnosis, clinical stage, treatments), and general health (National Cancer Institute [NCI] comorbidity index).

### Statistical analysis

Descriptive statistics were performed using univariate analysis with χ^2^test. To assess the potential independent association of variables with clinical outcome, multivariable logistic regression analysis (MVA) was used. Significance was defined with p-value (p) less than 0.05. Kaplan-Meier analyses (KMA) were used to compare overall survival (OS) by subgroups based on receipt of chemotherapy. All statistical analysis were conducted using SAS version 9.4 (SAS Institute Inc./Cary, NC).

## Results

**Figure 1:**
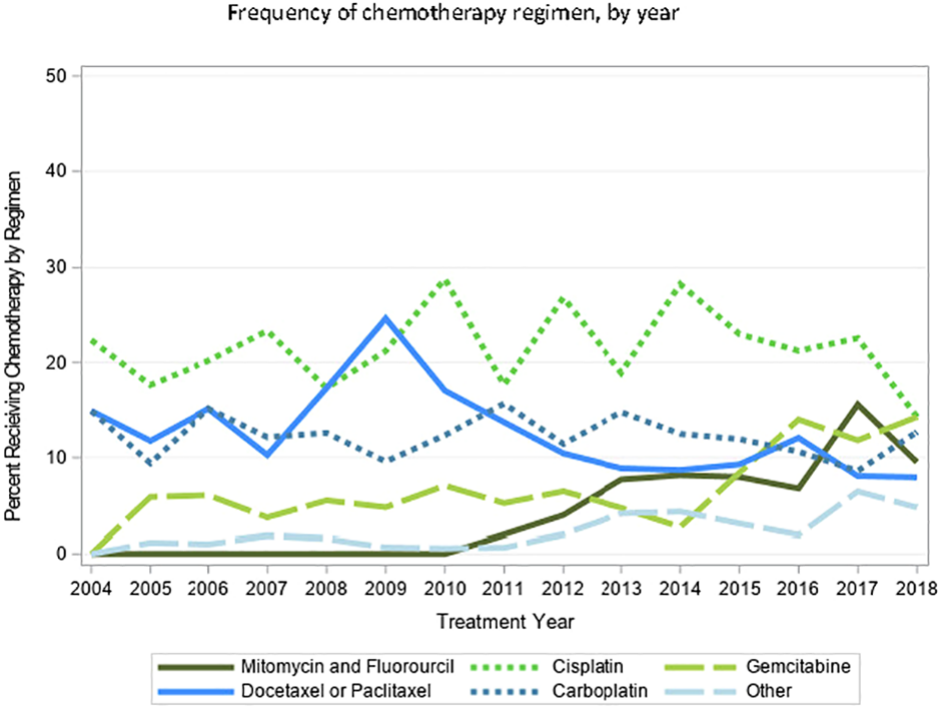
Temporal trends of choice of chemotherapeutic agent amongst patients with MIBC undergoing curative0intent RT, from 2004–2018.

A total of 17,648 patients with cT2-4N0/x M0/x BC were identified, 2,190 (12.4%) of whom received curative-intent primary RT based on the aforementioned definition, with a median age of 80 [interquartile range (IQR) 75, 85]. Of those receiving RT, 850 (38.8%) did not receive any concurrent chemotherapy with RT (**Table 1**). We found significant heterogeneity with respect to administered chemotherapeutic agent. The most common agents used were gemcitabine, cisplatin, carboplatin, or taxane (docetaxel or paclitaxel) (**Table 2**). In more recent years, the combination of mitomycin and fluorouracil, or gemcitabine alone, were increasingly used. Temporal trends and breakdown of agents are shown in **Figure 1** demonstrating slight increases in the utilization of gemcitabine and mitomycin + fluorouracil, and relative stability of other agents.

**Table 1:** Patient characteristics and rates of receiving concurrent chemotherapy.

Variable	Entire Cohort, n = 2190 (%)	Chemotherapy, n = 1340 (%)	No Chemotherapy, n = 850 (%)	p value
***Age (yrs)***				<0.0001
*65 – 79*	1001 (45.7%)	700 (52.2%)	301 (35.4%)	
*80+*	1189 (54.29%)	640 (47.8%)	549 (65.6%)	
***Race***				0.03
*Black*	109 (5.0%)	55 (4.1%)	54 (6.4%)	
*Hispanic*	84 (3.8%)	44 (3.3%)	40 (4.7%)	
*White*	1947 (88.9%)	1212 (90.4%)	735 (86.5%)	
*Other*	50 (2.3%)	29 (2.2%)	21 (2.5%)	
***Marital Status***				<0.001
*Married*	1214 (55.4%)	793 (59.2%)	421 (49.5%)	
*Single*	154 (7.0%)	87 (6.5%)	67 (7.9%)	
*Divorced*	733 (33.5%)	412 (30.7%)	321 (37.8%)	
*unknown*	89 (4.1%)	48 (3.6%)	41 (4.8%)	
***Clinical T-stage***				0.01
*cT2*	1855 (84.7%)	1157 (86.3%)	698 (82.1%)	
*cT3*	147 (6.7%)	86 (6.4%)	61 (7.2%)	
*cT4*	188 (4.1%)	97 (7.2%)	91 (10.7%)	
***Clinical Stage***				0.07
*II*	1769 (80.8%)	1106 (82.5%)	663 (78.0%)	
*III*	284 (13.0%)	159 (11.9%)	125 (14.7%)	
*IV*	27 (1.2%)	14 (1%)	13 (1.5%)	
***SES***				<0.001
*Lower*	656 (30.0%)	380 (28.4%)	276 (32.5%)	
*Middle*	373 (17.0%)	236 (17.6%)	137 (16.1%)	
*Higher*	966 (4.1%)	626 (46.7%)	340 (40.0%)	
*unknown*	195 (8.9%)	98 (7.3%)	97 (11.4%)	
***Rural status***				0.27
*Rural*	355 (16.2%)	208 (15.5%)	147 (17.3%)	
*Not rural*	1835 (83.8%)	1132 (84.5%)	703 (82.7%)	
***NCI Index***				<0.0001
*0*	736 (33.6%)	486 (36.3%)	250 (29.4%)	
*1*	1028 (46.9%)	638 (47.6%)	390 (45.9%)	
*2*	366 (16.7%)	192 (14.3%)	174 (20.5%)	
*3*	60 (2.7%)	24 (1.8%)	36 (4.2%)	

**Table 2:** Chemotherapeutic agent concurrently administered with primary RT.

Variable	Entire Cohort (n = 2190)
***Chemotherapy***	
*None*	850 (38.8%)
*Carboplatin*	265 (12.1%)
*Cisplatin*	489 (22.3%)
*Docetaxel or paclitaxel*	373 (12.4%)
*Gemcitabine*	154 (7.0%)
*Mitomycin + Fluorouracil*	106 (4.8%)
*Other*	54 (2.5%)

Receiving RT without concurrent radio-sensitizing chemotherapy was more common in those > 80 years of age (46.2%), black race (49.5%), Hispanic race (47.6%), single status (43.5%), divorced status (43.8%), cT3/T4 status (41.5% and 48.4%), lower SES (42.1%) (**Table 1**).

On MVA for receiving any concurrent chemotherapy, factors that were independently associated with decreased likelihood included increasing age (OR 0.93, CI 0.92 – 0.95), Hispanic race (compared with White, OR 0.62, CI 0.39 – 0.99), cT3 or T4 (compared with cT2, OR 0.70, CI 0.55 – 0.90), and lower NCI index (OR 0.60, CI 0.51 – 0.70) (p < 0.05). Variables independently associated with increased likelihood of receipt of chemotherapy were married status (OR 1.28, CI 1.06 – 1.54), higher SES (OR 1.31, CI 1.06 – 1.64), and later year of diagnosis (OR 1.09, CI 1.06 – 1.12). Black race was not independently associated with receipt of concurrent chemotherapy (OR 0.71, CI 0.47 – 1.08). Results are shown in **Table 3**A.

**Table 3:** Multivariable analysis for factors associated with (A) receiving concurrent chemotherapy with RT, and (B) for overall survival.

Variable	A			B		
	Odds Ratio	Confidence Interval	p value	Hazard Ratio	Confidence Interval	P value
***Age***	0.93	0.92 – 0.85	<0.001	1.01	1.00 – 1.02	0.02
*Race (vs. White)*						
*Black*	0.71	0.47 – 1.08	0.65	0.87	0.70 – 1.09	0.23
*Hispanic*	0.62	0.39 – 0.99	0.04	0.92	0.72 – 1.19	0.52
*Other*	0.80	0.44 – 1.44	0.89	1.24	0.91 – 1.69	0.37
*Married (vs. single)*	1.28	1.06 – 1.54	0.007	0.96	0.87-1.06	0.37
*SES (vs. lower)*						
*Higher*	1.31	1.06 – 1.64	0.01	0.93	0.83-1.04	0.21
*Middle*	1.17	0.89 – 1.54	0.88	1.02	0.88-1.18	0.82
***cT3/4 (vs cT2)***	0.70	0.55 – 0.90	0.01	1.24	1.09-1.41	<.001
***NCI Index***	0.60	0.51 – 0.70	<0.001	1.3	1.2-1.41	<.0001
***Year of diagnosis***	1.09	1.06 – 1.12	<0.001	0.63	0.57-0.69	<.0001
***Receipt of concurrent Chemotherapy (vs No Chemotherapy)***	–	–	–	0.97	0.96-0.99	<.001

**Figure 2:**
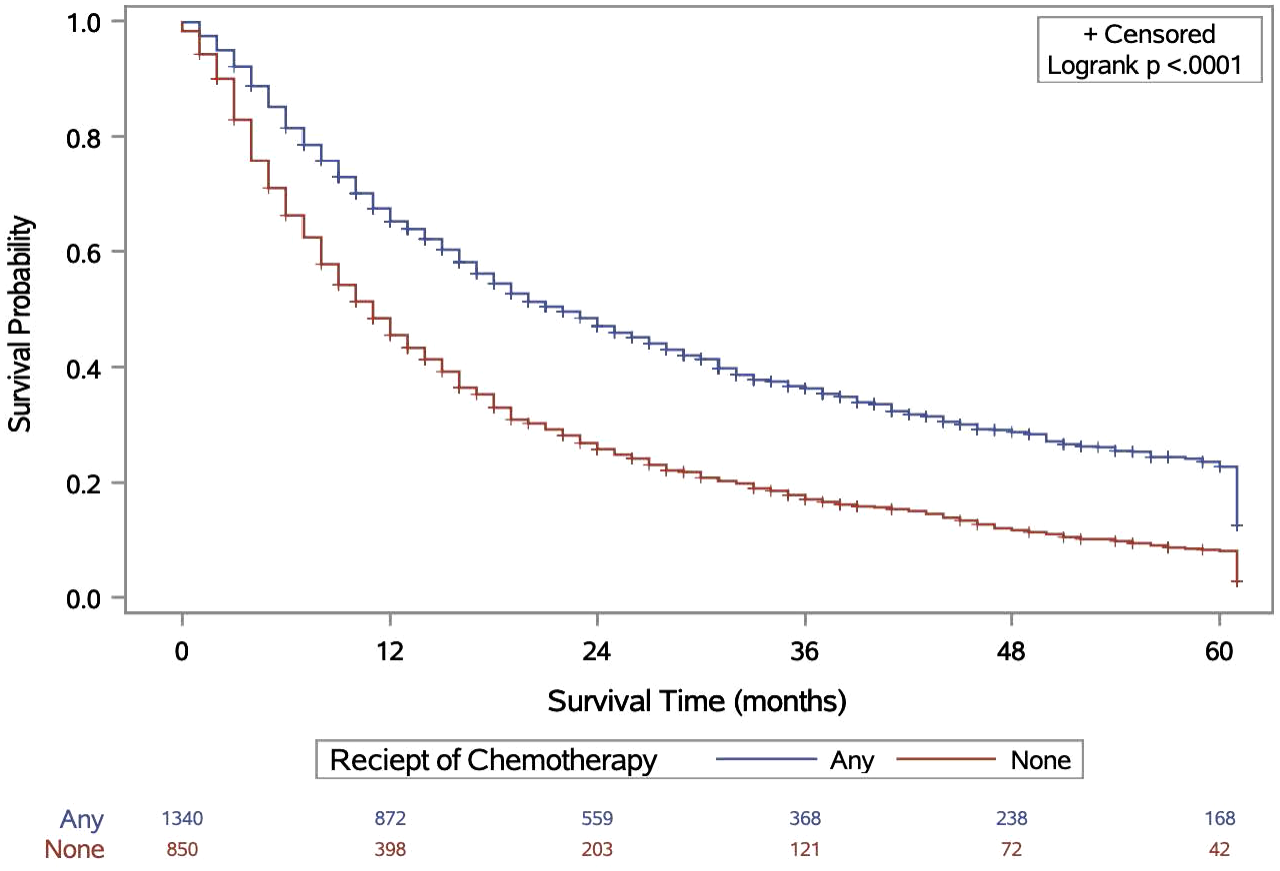
Kaplan–Meier analysis of surviaval in patients with MIBC treated with curative-intent RT, stratified by receipt of chemotherapy.

As shown in **Figure 2**, CRT was associated with significantly longer OS compared to RT alone (p < 0.0001). On MVA, receipt of chemotherapy was significantly associated with longer OS (HR 0.611, CI 0.55 – 0.68), shown in **Table 3**B. Race and SES were not associated with OS (p > 0.05).

## Discussion

The increasing adoption of bladder preservation for MIBC highlights a strong interest in bladder-sparing therapeutic options amongst both providers, advocacy groups, e.g. Bladder Cancer Advocacy Network, and patients (17). Patterns of chemotherapy administration within CRT remain poorly described, however, and evidence suggests underutilization of any chemotherapy with RT (9, 14, 15). We report an analysis of the national SEER-Medicare database and found that 39% of patients with localized BC undergoing primary curative-intent RT receive no concurrent chemotherapy.

The addition of chemotherapy to RT in MIBC is supported by level 1 evidence and is the current standard of care (1, 10–13, 18, 19). However, widespread adoption of chemotherapy with curative-intent treatment in MIBC has been slow. Parallels have been seen as well in patients that are surgically managed. NAC prior to RC improves OS based on level 1 evidence, yet has historically also been underutilized, though recent studies document increasing adoption (20–23). Less is known regarding the use of radio-sensitizing chemotherapy concurrently with RT. Xiang et al. found that 42% of MIBC patients receiving radiation within the National Cancer Database (NCDB) received no concurrent chemotherapy (14). Data on specific chemotherapeutic agents are not available in NCDB. Similarly, analysis of multi-institutional cohorts from high-volume centers report 47-56% of patients undergo curative-intent radiation alone, and are not given concurrent chemotherapy (15). Analysis of the Ontario Cancer Registry found 36-48% of patients received CRT, with the rest receiving RT alone (9). Our findings within SEER-Medicare are consistent with previous reports and provide further evidence of this underutilization on a national scale.

One potential cause for chemotherapy underutilization might be concern regarding tolerability/toxicity. We and others (9, 14) have noted that older age, poor performance status, frailty and higher comorbidity indices have been associated with foregoing chemotherapy. It is important to note, however, the high tolerability of concurrent chemoradiation in the trial setting (10, 24). In their landmark 2012 trial, James et al. for example, found only a marginal difference in grade 3-5 toxicity in the chemoradiotherapy group (receiving fluorouracil and mitomycin) compared with radiotherapy alone (36% vs 27.5% respectively, p=0.07) and no differences seen at 1 or 2 years (10). Similarly, in a phase 2 study of gemcitabine and RT, Choudhury et al. found only four of 50 patients (8%) discontinued chemotherapy due to side effects (24). We found an increasing rate of chemotherapy utilization over time, perhaps reflecting a delayed implementation of these findings. Still, the underutilization of well-tolerated radio-sensitizing chemotherapy represents an important target for quality improvement efforts in the bladder-sparing management of MIBC.

There was significant heterogeneity among chemotherapy agents utilized. In this group, 44% of patients received chemotherapy that falls outside of currently recommended regimen - cisplatin, gemcitabine, fluorouracil + mitomycin, and cisplatin + fluorouracil or paclitaxel - and many were treated with agents that are not supported for radio-sensitization. Carboplatin alone was used in 20% of patients, for example, despite data demonstrating inferior outcomes and increased toxicity with this agent. In a review of long-term institutional data, for example, receipt of carboplatin with RT was associated with poorer complete response on restaging TURBT as well as OS compared with cisplatin (11). Kumar et al. reviewed data from the veterans administration and found patients that received non-preferred chemotherapy with radiation (which includes carboplatin) had significantly shorter OS and bladder cancer specific survival (4). Our findings in this national cohort are consistent with previous reports from multi-institutional data, which similarly demonstrate significant heterogeneity, and high rates of use of unvalidated regimens (15). Ghate et al. reported the Canadian experience demonstrating 31% of patients who received chemotherapy were given carboplatin (9). The relative standardization of chemotherapeutic agent is a second important target for quality improvement with curative-intent radiation for MIBC. We consider cisplatin to be first line as a radiosensitizing regimen, and advocate for fluorouracil + mitomycin or single-agent gemcitabine as alternatives in cisplatin-ineligible patients.

Hispanic race and lower SES were independently associated with chemotherapy underutilization in our study. Significant disparities across races/ethnicities have long been described in urologic oncology (25–27). Within MIBC specifically, Washington et al. report a significantly lower proportion of standard of care treatment amongst Black compared to White patients (37% vs 43% respectively, OR 0.72 [0.67-0.79], p<0.001), and Fang et al. found shorter OS amongst Black patients (28, 29). In our analysis, Black race was not associated with chemotherapy use, nor was it associated with shorter OS among patients receiving RT. Less is known regarding the disparities associated with Hispanic race within bladder cancer. Recent analysis of the SEER-Medicare database in metastatic patients with bladder cancer found the poorest cancer specific survival in Hispanic patients compared to their Black, White, and Asian counterparts (30). In non-MIBC, Noel et al. reported underutilization of adjuvant intravesical therapy in Hispanic and Asian patients, relative to other racial groups (31). The etiologies of racial and ethnic disparities in cancer care are complex and multifactorial. Prostate cancer health equity literature has demonstrated the normalization of outcome differentials by race within equal access systems like Veterans Health Administration and within clinical trials (32, 33). Amongst patients with bladder cancer, equal access to healthcare systems have been associated with improved, yet persistently detectable, racial disparities (34). These findings suggest a promising avenue for addressing outcome disparities among races and ethnic groups (33). Moving forward, efforts to minimize the underuse of radio-sensitizing chemotherapy should focus on the most vulnerable patient groups who are disproportionately impacted.

In our study, patients receiving chemotherapy had longer OS, and chemotherapy administration was an independent factor associated with longer survival, after accounting for age, clinical stage, health, SES, and other available confounders. Association between receipt of chemotherapy and longer OS has been noted in other retrospective studies. Williams et al. report longer OS for patients receiving cisplatin or 5-FU+mitomycin-c compared to other regimens (35). Others have found chemotherapy administration to be associated with improved rates of treatment completion, which has been associated with longer OS (36). Still, it is important to note that randomized clinical trials have not demonstrated a significant OS benefit with the addition of concurrent chemotherapy to primary radiation, only improvements in locoregional recurrence rates and bladder preservation rates (10–13, 18, 19).

There are several inherent limitations worth highlighting in the present study. Firstly, this is a retrospective analysis of a large national database (with lack of randomization), limited to the accuracy and robustness of available data. Errors in coding and uncoded clinical details (missing data) were inaccessible to the authors, and so could not be accounted for in our analysis. Moreover, the impact of several important clinical and pathologic factors, such as histologic variants, were not evaluated in this study. Great care must be taken in interpretating outcomes data given the inability to account for all potential unmeasured confounders and selection bias. Additionally, the intent of radiation – curative versus palliative – was not available in the database, and this study defined curative-intent radiation courses as those receiving 11 fractions or more. This cut-off was selected based on the most common palliative regimens of 21Gy in 3 fractions or 30Gy for 10 fractions (1), but we recognize that aggressive palliative regimen of >10 fractions are occasionally administered. This may have included patients who received a more extended course of palliative-intent radiotherapy, who may be less fit and less likely to be candidates for concurrent chemotherapy, potentially inflating the underutilization rates of chemotherapy for curative-intent treatment. Moreover, differential access to effective subsequent therapies may impact the OS analysis.

In conclusion, a large proportion of patients with MIBC undergoing curative-intent primary RT were not given concurrent chemotherapy, and many received suboptimal agents. Optimizing concurrent chemotherapy administration rates represents a key area of opportunity for quality improvement for patients with localized MIBC.

## Data availability statement

Publicly available datasets were analyzed in this study. This data can be found here: https://healthcaredelivery.cancer.gov/seermedicare/.

## Author contributions

FG: Writing – review & editing, Writing – original draft, Validation, Formal analysis, Data curation, Conceptualization. SH: Writing – review & editing, Validation, Investigation, Data curation, Conceptualization. EK: Writing – review & editing. JC: Writing – review & editing. EW: Writing – review & editing, Supervision, Methodology, Investigation. JL: Writing – review & editing, Methodology. JZ: Writing – review & editing, Supervision, Methodology. PG: Writing – review & editing, Conceptualization. JH: Writing – review & editing, Investigation, Conceptualization. AH: Writing – review & editing, Methodology, Conceptualization. RM: Writing – review & editing, Supervision, Methodology. JW: Writing – review & editing, Supervision, Methodology, Funding acquisition, Formal analysis, Data curation, Conceptualization.
