# Supplementary material for: Patterns of chemotherapy use with primary radiotherapy for localized bladder cancer in patients 65 or older
Source: Front Oncol. 2024 May 15;14:1341655. doi: 10.3389/fonc.2024.1341655 (PMC11133586; doi:10.3389/fonc.2024.1341655)
Supplement: Supplementary file 1 [file Table_1.docx]

Supplementary Table – ICD and CPT codes for radiation and chemotherapy

|  | ICD Procedure Codes | CPT Codes |
| --- | --- | --- |
| Radiation Therapy | 92.20–92.27, 92.29, 92.30-92.39, 92.4, 92.41 | 77371–77373, 77401–77525, 77761–77799, G0174, G0251, G0339, G0340, G0173, 0082T, 61793, 0182T |
| Chemotherapy |  | J6360, J9000, J9001, J9060, J9062, J9201, J9250, J9260, J9000, J9010, J9250, J9260, J9360, J9070, J9080, J9090, J9091, J9092, J9093, J9094,  J9095, J9096, J9097, J9098, J9201, J9265, J9170, J9208  J9999, J9045, J9271, J9022, J9023, J9025, J9299 |
